# Recurrent thymoma-associated PRCA with persistent agranulocytosis: a case report and therapeutic considerations

**DOI:** 10.3389/fonc.2026.1930291

**Published:** 2026-09-28

**Authors:** Antuo Wang, Yu Xia, Mengyuan Xin, Liang Song, Bin Jiang, Mo Shi, Zhongwei Xin

**Affiliations:** 1 Department of Thoracic Surgery, Shandong Provincial Hospital Affiliated to Shandong First Medical University, Jinan, Shandong, China; 2 Medical Science and Technology Innovation Center, Shandong First Medical University, Jinan, Shandong, China; 3 School of Pharmacy, Shandong First Medical University, Jinan, Shandong, China

**Keywords:** agranulocytosis, cyclosporine A, immune-mediated hematopoietic injury, pure red cell aplasia, thymectomy, thymoma

## Abstract

We report a case of recurrent type A thymoma accompanied by pure red cell aplasia (PRCA) and agranulocytosis. Following local recurrence after thymoma resection, the patient gradually developed anemia and leukopenia as the lesion progressed; bone marrow examination showed marked suppression of erythropoiesis and reduced granulopoiesis. Taken together with the peripheral blood findings, these results suggested thymoma-associated immune-mediated hematopoietic injury. Following multidisciplinary evaluation, the patient underwent thoracoscopic resection of the recurrent lesion, and pathological examination confirmed type A thymoma with negative surgical margins. Postoperative follow-up showed gradual recovery of erythropoiesis and achievement of transfusion independence, whereas agranulocytosis persisted; only after regular cyclosporine A treatment and increased drug exposure did the white blood cell and neutrophil counts gradually rise. Based on this case and previous studies, thymoma resection may reduce immune attack on the bone marrow and create conditions favorable for hematopoietic recovery by removing the source of continued generation and export of abnormal T cells; however, abnormal T cells that have already entered the peripheral blood or bone marrow and continue to survive may perpetuate immune-mediated injury. Therefore, blood counts should be monitored serially after surgery, and standardized adjunctive immunosuppressive therapy should be instituted when necessary. During treatment, close attention should also be paid to medication adherence, therapeutic drug concentrations, and monitoring of hepatic and renal function to balance efficacy and safety.

## 1 Introduction

Thymoma may be associated with a variety of hematologic paraneoplastic syndromes, including pure red cell aplasia (PRCA), agranulocytosis, and acute leukemia (1–4). Although these complications are uncommon, they may lead to severe anemia, serious infections, and persistent hematopoietic dysfunction, thereby substantially affecting patient outcomes (1–4).

Thymectomy and immunosuppressive therapy are important therapeutic approaches for thymoma-associated bone marrow failure (2–4). However, the immunologic mechanisms by which these treatments improve hematopoietic function remain incompletely understood. We therefore propose a “thymus–peripheral blood–bone marrow axis” hypothesis as a unified mechanistic framework for explaining thymoma-associated immune-mediated hematopoietic injury and the effects of related therapies. According to this hypothesis, thymectomy may alleviate immune-mediated injury to bone marrow hematopoietic cells by eliminating the abnormal thymic microenvironment, reducing the continuous generation and peripheral egress of autoreactive T cells, and facilitating the restoration of regulatory T-cell (Treg)-mediated immune tolerance (5, 6). Immunosuppressive agents, particularly cyclosporine A (CsA), may further attenuate immune-mediated hematopoietic injury and promote hematopoietic recovery by suppressing the persistent activation and clonal expansion of aberrant T cells (2, 4, 6). In patients with persistent hematologic abnormalities after thymectomy, the continued survival or expansion of T-cell large granular lymphocyte (T-LGL)-like aberrant CD8^+^ T-cell clones may represent one of the mechanisms underlying persistent immune-mediated injury and impaired myelopoiesis (7–9).

In this article, we examine this hypothesis in the context of a patient with recurrent thymoma, focusing on dynamic perioperative changes in hematopoietic function and the response to CsA therapy. These clinical observations provide indirect support for the proposed mechanistic framework and suggest that the integration of thymectomy with subsequent immunosuppressive therapy may offer complementary and potentially synergistic therapeutic benefits.

## 2 Case presentation

The overall clinical course is summarized in **Supplementary Figure S1**.

### 2.1 Medical history and clinical course

The patient was a 68-year-old woman. More than 10 years earlier, an anterior mediastinal mass had been detected during a routine physical examination and surgically resected; postoperative pathological examination established a diagnosis of thymoma, after which she underwent regular follow-up. In 2020, follow-up chest computed tomography (CT) revealed an anterior mediastinal lesion, which was considered postoperative recurrence of the thymoma, and imaging surveillance was continued. During follow-up, she gradually developed anemia and leukopenia of unknown cause, for which no specific treatment was administered.

In February 2025, because of progressive worsening of the anemia and leukopenia, the patient underwent bone marrow cytomorphologic examination in the Department of Hematology. A bone marrow smear obtained on February 24 showed hypocellular marrow, decreased granulopoiesis with mild dysplasia, and absent erythropoiesis (**Figure 1A**, left). After hematopoietic support, repeat bone marrow cytomorphology on February 26 showed active granulopoiesis and megakaryopoiesis, whereas erythropoiesis remained markedly reduced (**Figure 1A**, right). Based on the medical history, peripheral blood counts, and bone marrow findings, the clinical impression was PRCA complicated by agranulocytosis, and these hematologic abnormalities were considered possibly attributable to a paraneoplastic syndrome associated with recurrent thymoma.

**Figure 1:**
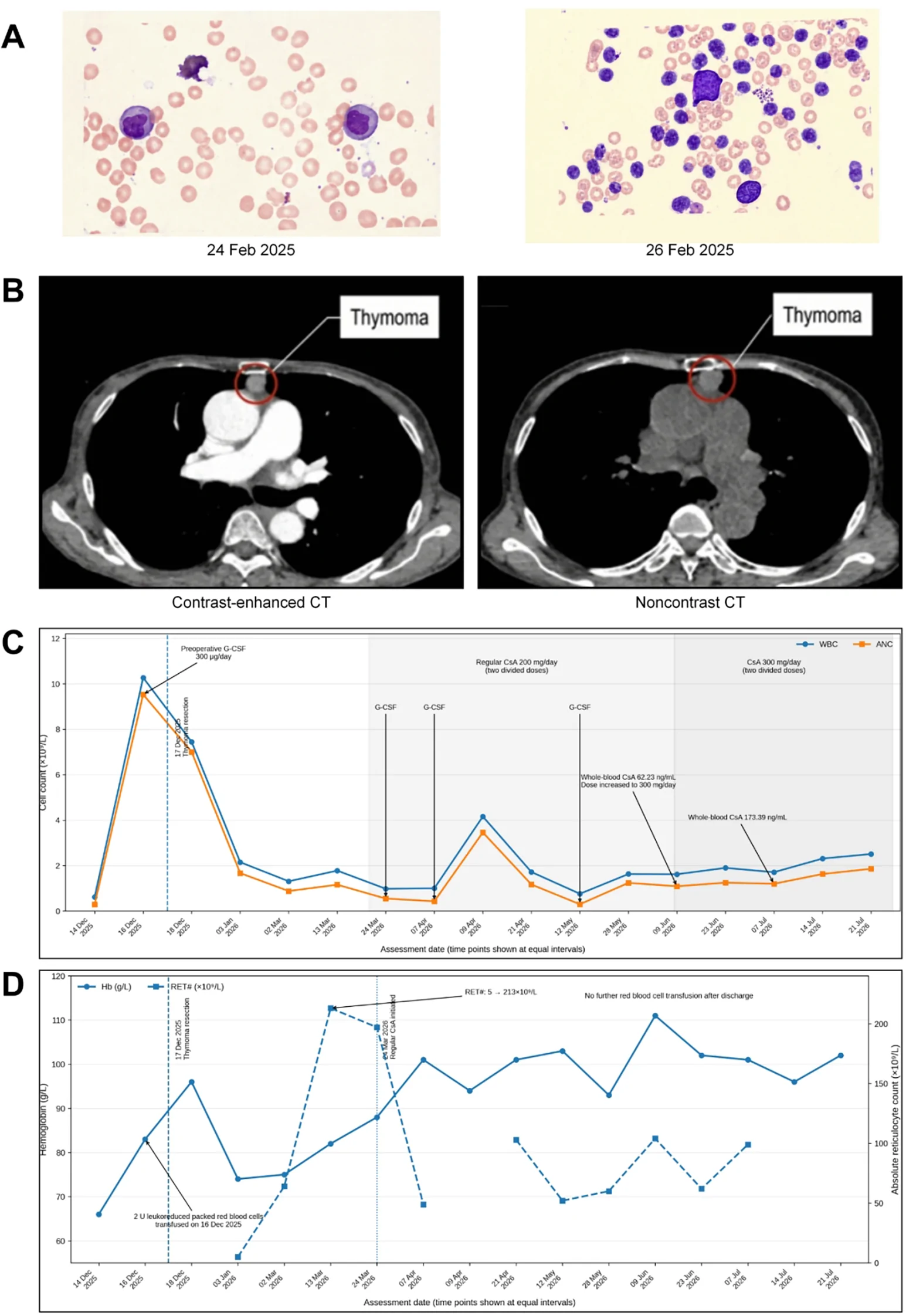
Clinical, morphologic, imaging, and hematologic findings in the present case. **(A)** Bone marrow smears obtained on February 24 and February 26, 2025. **(B)** Contrast-enhanced and noncontrast chest CT demonstrating the recurrent anterior mediastinal lesion consistent with thymoma. **(C)** Serial changes in white blood cell count (WBC) and absolute neutrophil count (ANC) during follow-up, together with granulocyte colony-stimulating factor (G-CSF) administration and cyclosporine A (CsA) treatment/exposure. **(D)** Serial changes in hemoglobin (Hb) and absolute reticulocyte count during follow-up.

Thereafter, the patient intermittently received leukoreduced packed red blood cell transfusions, hematopoietic support, and oral immunosuppressive treatment with CsA. CsA was initially prescribed at 150 mg/day in two divided doses, with a plan to gradually adjust the dose to 200–300 mg/day according to efficacy and tolerability. However, medication adherence was poor, and the patient repeatedly reduced the dose on her own; the highest dose actually taken was only 75 mg/day, with no clear improvement in either anemia or neutropenia.

In December 2025, the patient presented to the Department of Thoracic Surgery with a foreign-body sensation in the throat, paroxysmal cough, occasional dysphagia, and dull pain in the upper anterior chest. Chest CT on December 1 showed enlargement of the recurrent anterior mediastinal lesion compared with previous imaging, suggesting progression of recurrent thymoma. Because the lesion continued to enlarge and the hematologic abnormalities described above might have been thymoma-related, surgical resection was planned after multidisciplinary evaluation, and the patient was admitted on December 14.

### 2.2 Past medical history

The patient had a 3-year history of myasthenia gravis (MG). Her symptoms improved after glucocorticoid therapy, which was gradually tapered and discontinued, and the related symptoms did not recur after treatment withdrawal. Hyperglycemia developed during glucocorticoid therapy but stabilized after glucocorticoid discontinuation.

She had a history of coronary artery disease and a prior myocardial infarction dating back more than 1 year and had previously received indobufen and clopidogrel, which were discontinued approximately 20 days before admission. She also had long-standing hypertension, chronic gastritis, and a history of hepatitis in childhood. She was allergic to penicillins and cephalosporins. She had no history of smoking or alcohol consumption and no known exposure to specific toxins. Her family history was notable for hypertension, and she denied a family history of malignancy.

### 2.3 Assessment at admission

At admission, the patient’s temperature was 36.2 °C, pulse rate was 81 beats/min, respiratory rate was 20 breaths/min, blood pressure was 128/77 mmHg, and body weight was 46 kg. A murmur was audible over the aortic valve area; the remainder of the physical examination revealed no obvious abnormalities.

Preoperative complete blood count showed a white blood cell count (WBC) of 0.62×10^9^/L, hemoglobin (Hb) of 66 g/L, platelet count (PLT) of 123×10^9^/L, and absolute neutrophil count (ANC) of 0.29×10^9^/L, indicating agranulocytosis and marked anemia. Coagulation studies were unremarkable. Liver function tests showed an aspartate aminotransferase (AST) level of 65 U/L and a γ-glutamyl transferase (GGT) level of 62 U/L.

After hematology consultation, the patient received 300 μg of granulocyte colony-stimulating factor (G-CSF) and 2 U of leukoreduced packed red blood cells before surgery. Repeat complete blood count after treatment showed a WBC of 10.27×10^9^/L, Hb of 83 g/L, PLT of 151×10^9^/L, and ANC of 9.53×10^9^/L.

Noncontrast and contrast-enhanced chest CT revealed a nodular soft-tissue attenuation lesion in the anterior mediastinum with well-defined margins, mildly heterogeneous internal attenuation, and marked enhancement (**Figure 1B**). In view of the previous surgical history, local postoperative recurrence of thymoma was considered. Scattered small nodules in both lungs showed no obvious interval change, and no pleural effusion was present.

Echocardiography showed mild enlargement of the left atrium and left ventricle, a left ventricular ejection fraction of approximately 49%, mild aortic regurgitation, and mild dilatation of the ascending aorta. Coronary CT angiography showed no definite stenosis, with a Coronary Artery Disease–Reporting and Data System (CAD-RADS) category of 0. Electrocardiography showed sinus rhythm, complete right bundle branch block, and abnormal Q waves and ST-T changes in leads II, III, and aVF.

### 2.4 Surgery and pathology

Following multidisciplinary discussion involving thoracic surgery, hematology, and anesthesiology, the anterior mediastinal lesion was considered localized and resectable, and the patient was deemed fit for surgery after preoperative supportive treatment. On December 17, 2025, the recurrent anterior mediastinal lesion was resected under general anesthesia through a left thoracoscopic approach. No pleural effusion or definite pleural dissemination was observed intraoperatively. A nodular lesion adjacent to the ascending aorta in the anterior mediastinum was identified and resected.

Postoperative pathological examination established a diagnosis of type A thymoma. The tumor measured approximately 2.2 cm×1.6 cm×1.3 cm, and the resection margins were negative. Immunohistochemical staining showed positivity for p63 and CK19; p53 was weakly positive in approximately 30% of cells, and the Ki-67 proliferation index was approximately 5%-10%. CK (AE1/AE3) was negative, CD3 was focally positive, and CD20, CD5, CD117, CD1a, and TdT were negative.

Because of perioperative considerations, CsA and other immunosuppressive agents were withheld during the perioperative period. Postoperative recovery was uneventful, with no significant bleeding, infection, or other complications. Complete blood count on postoperative day 1 showed a WBC of 7.44×10^9^/L, Hb of 96 g/L, PLT of 142×10^9^/L, and ANC of 7.00×10^9^/L; liver function tests showed an AST level of 71 U/L and a GGT level of 57 U/L. Because the patient had received G-CSF before surgery, the perioperative increase in granulocyte counts could not be taken as evidence that granulopoiesis had recovered. The chest drain was removed on postoperative day 2, and the patient was discharged on December 20.

### 2.5 Follow-up and outcomes

After discharge, complete blood counts, absolute reticulocyte counts, and hepatic and renal function were monitored regularly. During follow-up, the patient received no further red blood cell transfusions, and postoperative chest CT showed no evidence of thymoma recurrence. The absolute peripheral blood reticulocyte count increased from 5.0×10^9^/L on January 3, 2026, to 213.0×10^9^/L on March 13 and subsequently remained at 48.9-104.0×10^9^/L. The Hb level gradually increased from 74 g/L and remained at 93–111 g/L after April, indicating sustained recovery of erythropoiesis and achievement of transfusion independence (**Figure 1D**).

However, during early follow-up, the peripheral blood WBC and ANC remained persistently low and fell to levels consistent with agranulocytosis at some time points. After each administration of G-CSF, the WBC and ANC increased only transiently and then declined again. Beginning on March 24, 2026, the patient regularly took oral CsA at 200 mg/day in two divided doses, as advised by the hematology team; however, the WBC and ANC still showed no clear improvement, and intermittent G-CSF remained necessary. On June 9, the whole-blood CsA concentration was 62.23 ng/mL, indicating low drug exposure; the CsA dose was therefore increased to 300 mg/day in two divided doses. On July 7, the whole-blood CsA concentration had increased to 173.39 ng/mL. Thereafter, without further G-CSF administration, the WBC and ANC showed an overall upward trend, increasing from 1.62×10^9^/L and 1.09×10^9^/L, respectively, on June 9 to 2.51×10^9^/L and 1.86×10^9^/L, respectively, on July 21, suggesting that granulopoietic recovery may have been associated with increased CsA exposure (**Figure 1C**).

Given the patient’s advanced age, history of hepatitis, and need for long-term CsA therapy, hepatic and renal function were closely monitored during follow-up. She also received oral bicyclol 50 mg three times daily and silibinin meglumine 100 mg three times daily. Although liver function indices fluctuated to some extent during follow-up, they remained stable overall, without progressive deterioration. Renal function indices also showed mild fluctuations after adjustment of the CsA dose, but no progressive decline in renal function was observed.

The detailed longitudinal follow-up data are provided in **Supplementary Table S1**.

## 3 Discussion

### 3.1 Potential immune mechanisms underlying thymoma-associated hematopoietic injury in the bone marrow

Current evidence suggests that thymoma-associated disruption of immune tolerance may contribute to immune-mediated bone marrow injury (5, 10–12). Studies have shown that, in the normal thymus, functionally distinct subsets of medullary thymic epithelial cells (mTECs) cooperate to maintain central T-cell tolerance: CCL21^+^ mTECs guide positively selected but not yet mature T cells into the medulla, whereas Aire^+^ and Fezf2^+^ mTECs express and present self-antigens, thereby promoting the deletion of autoreactive T cells and the generation of Tregs (10). Thymomas may exhibit AIRE deficiency and mTEC immaturity (5, 11, 12). For example, AIRE protein may be undetectable in the tumor epithelial cells of type A thymomas, and thymic epithelial tumors dominated by cortical thymic epithelial cell (cTEC)-like cells may also lack AIRE gene expression (11, 12). In addition, thymomas may be accompanied by disruption of corticomedullary compartmentalization, defective expression of T-cell chemokines and molecules involved in positive and negative selection, and a reduced proportion of Tregs (5, 11, 12). Consequently, autoreactive T cells that should have been eliminated may survive, forming the basis of thymoma-associated immune injury (5, 11, 12) (**Figure 2A**).

**Figure 2:**
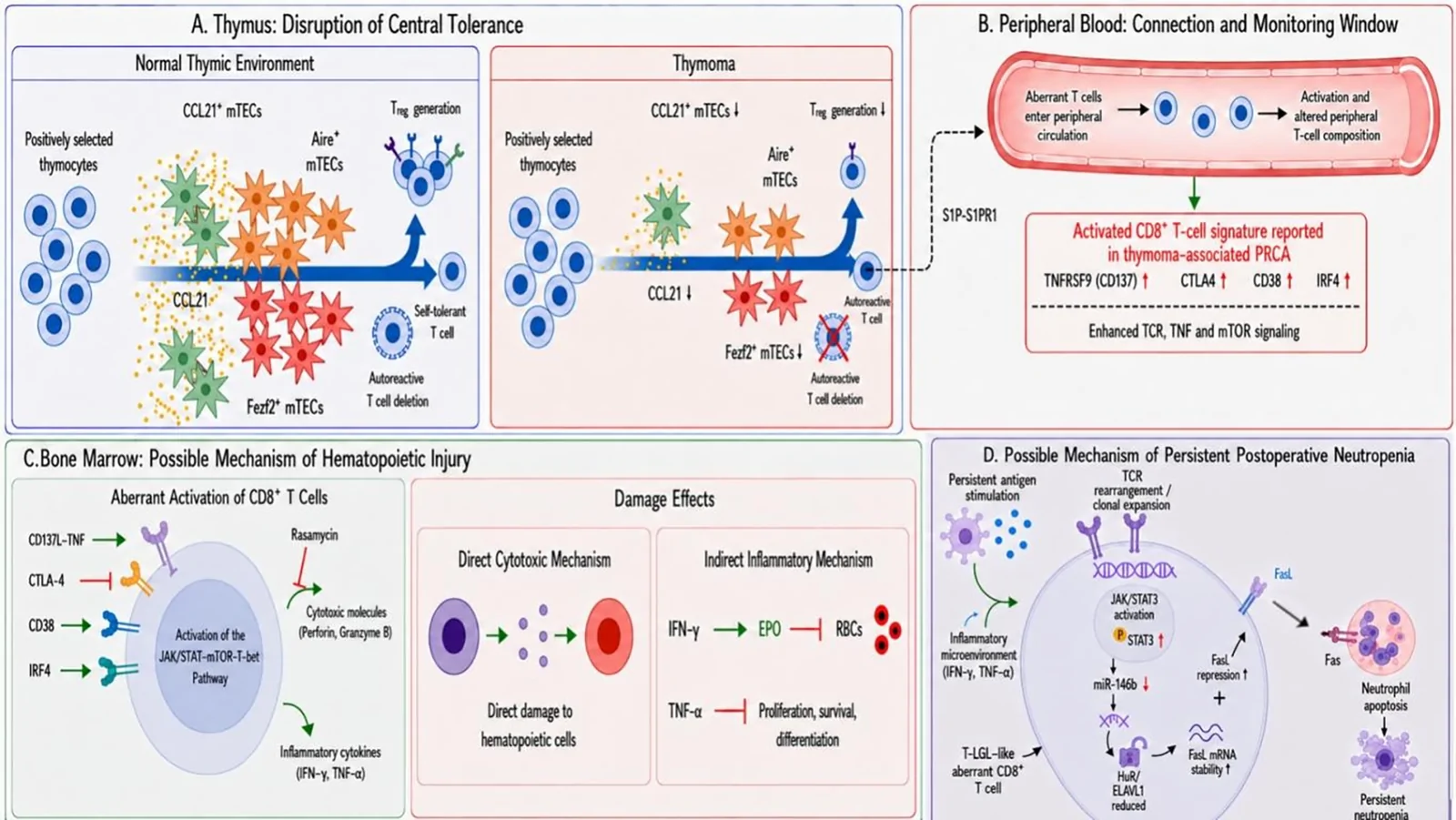
Proposed immune mechanisms underlying thymoma-associated hematopoietic injury and persistent postoperative neutropenia. **(A)** Disruption of central tolerance in the thymus. **(B)** Peripheral blood as a connection and monitoring window for abnormal T-cell export and activation. **(C)** Possible mechanisms of immune-mediated hematopoietic injury in the bone marrow. **(D)** Possible mechanism of persistent postoperative neutropenia after thymoma resection. This schematic is a hypothesis-based summary derived from the literature and the present case.

No study has directly clarified how these abnormal T cells enter the circulation, nor has it been established whether they leave the thymus through the physiological sphingosine-1-phosphate (S1P)-sphingosine-1-phosphate receptor 1 (S1PR1) axis. However, peripheral blood studies indicate that thymomas can continuously export abnormal T cells and reshape the peripheral T-cell repertoire (13, 14). For example, the proportions of CD45RA^+^CD8^+^ T cells and IL-8^+^CD31^+^ naive CD4^+^ and CD8^+^ T cells are increased and decline after thymectomy (13, 14). Peripheral blood CD8^+^ T cells from patients with thymoma-associated PRCA also exhibit a pronounced activated transcriptional profile, characterized by upregulation of genes such as TNFRSF9, CTLA4, IRF4, and CD38, together with enhanced activity of the T-cell receptor (TCR), tumor necrosis factor (TNF), and mechanistic target of rapamycin (mTOR) pathways (6). These findings suggest that peripheral blood may be an important route linking the thymus and bone marrow and may also serve as a window for monitoring the export and activation status of abnormal T cells (**Figure 2B**).

Abnormally activated CD8^+^ T cells can be detected in both the peripheral blood and bone marrow of patients with thymoma-associated PRCA (6). Given the increased CXCR4 expression in a subset of mature T cells within thymomas and the ability of the CXCR4-CXCL12 axis to promote recruitment of pathogenic T cells to the bone marrow in models of aplastic anemia, it may be hypothesized that, after entering the peripheral circulation, these cells home to and are retained in the bone marrow through this axis (6, 15); however, this possibility requires further verification. Once in the bone marrow, abnormal CD8^+^ T cells may impair hematopoiesis through a combination of cytotoxic responses and inflammatory suppression. Studies have found marked reductions in burst-forming unit-erythroid (BFU-E), colony-forming unit-erythroid (CFU-E), and erythroblasts in the bone marrow of patients with thymoma-associated PRCA, suggesting a selective block in the erythroid developmental sequence (6). Concomitantly, CD8^+^ T-cell numbers were increased, with enhanced expression of perforin, granzyme B, interferon-γ (IFN-γ), and TNF-α (6, 16). Studies of acquired PRCA have also shown activation of the phosphoinositide 3-kinase (PI3K)-AKT-mTOR/T-bet pathway in bone marrow CD8^+^ T cells, and rapamycin can reduce perforin and granzyme B expression (16). These observations suggest that abnormal CD8^+^ T cells may directly damage erythroid progenitors through the perforin-granzyme B pathway and may also cause sustained suppression of hematopoiesis through IFN-γ and TNF-α (6, 16). Specifically, IFN-γ can antagonize the action of erythropoietin, whereas TNF-related signaling can inhibit the proliferation, survival, and differentiation of erythroid progenitors (6, 16). Abnormalities of CD8^+^ T cells in the peripheral blood and bone marrow, together with recovery of granulopoiesis after immunosuppression, have also been reported in thymoma-associated pure white cell aplasia, suggesting that a similar mechanism may cause agranulocytosis by suppressing granulocyte-macrophage colony-forming units (CFU-GM) and subsequent granulocytic differentiation (17) (**Figure 2C**).

On the basis of the evidence above, we propose a “thymus-peripheral blood-bone marrow axis” hypothesis: impaired central immune tolerance within the thymoma permits the generation of autoreactive T cells and their entry into the peripheral circulation, after which some of these cells further home to the bone marrow and cause immune-mediated hematopoietic injury (**Figures 2A–C**). In the present case, erythropoiesis gradually recovered after resection of the recurrent thymoma, and neutropenia subsequently improved after regular CsA treatment with increased drug exposure, providing indirect clinical support for this hypothesis.

It is noteworthy that the patient had a history of MG. MG is one of the most common autoimmune manifestations associated with thymoma, and its presence suggests that this patient may have had a broader thymoma-related disturbance of immune tolerance. Therefore, the PRCA and agranulocytosis observed in this case may not have been driven solely by abnormalities along the proposed “thymus–peripheral blood–bone marrow axis,” but may instead, together with MG, reflect a broader state of thymoma-associated systemic autoimmune dysregulation. However, the clinical course provides some distinction between these manifestations: the patient’s MG had achieved clinical remission after previous glucocorticoid therapy and did not recur after treatment discontinuation, whereas the anemia and leukopenia progressively worsened during the progression of recurrent thymoma and subsequently improved after tumor resection and regular CsA therapy, respectively. Thus, active MG itself appears insufficient to directly account for the dynamic changes in the hematologic abnormalities observed in this case. Nevertheless, because comprehensive immunologic evaluation of the different autoimmune effector pathways was not performed, the relative contributions of MG-associated immune abnormalities, the thymoma itself, and other aberrant T-cell responses to bone marrow injury cannot be fully distinguished.

### 3.2 Immune-mediated hematopoietic injury may persist after thymoma resection

After thymoma resection in the present case, the absolute reticulocyte count and Hb level continued to increase without further red blood cell transfusions, indicating gradual recovery of erythropoiesis. However, the WBC and ANC repeatedly declined and reached the range of agranulocytosis at some time points. This may have reflected slower recovery of granulopoiesis than of erythropoiesis, or it may have indicated that immune-mediated injury persisted after surgery. During follow-up, each administration of G-CSF produced only a transient increase in the WBC and ANC, followed by another decline. Both counts began to increase steadily only after the patient took CsA consistently and drug exposure was increased, further supporting the persistence of postoperative immune-mediated injury.

Previous studies suggest that persistent antigenic stimulation and an inflammatory microenvironment can confer a survival advantage on abnormal cytotoxic T cells and promote the continued expansion of cellular clones with specific TCR rearrangements (7–9). Persistent activation of the Janus kinase/signal transducer and activator of transcription (JAK/STAT) pathway, particularly STAT3 abnormalities, may further enable these cells to evade apoptosis (7, 9). Populations characterized by clonal expansion of cytotoxic T cells and STAT3 abnormalities but not yet meeting the diagnostic criteria for typical T-cell large granular lymphocytic leukemia (T-LGLL) are often referred to as “T-LGL-like” cytotoxic T-cell clones (7, 9). Oligoclonal effector CD8^+^ T cells associated with gain-of-function STAT3 alterations have likewise been termed “T-LGL-like effector CD8^+^ T cells” (7). Similar cells have been reported in thymoma-associated immune-mediated multilineage cytopenia (8). Through sustained STAT3 activation, these cells may downregulate miR-146b and relieve its suppression of HuR/ELAVL1, thereby increasing Fas ligand (FasL) mRNA stability and FasL expression and promoting neutrophil apoptosis (18). Therefore, these cells may survive for prolonged periods after thymectomy, causing refractory agranulocytosis or even manifesting as T-LGLL (**Figure 2D**).

A 2025 immunogenomic study showed that 77% of patients with T-LGLL had lymphopenia and/or hypogammaglobulinemia, and some patients responded to CsA therapy (9). The present patient had long-standing lymphopenia and low serum globulin levels and responded to CsA, partially mirroring these reported features. Because lymphopenia, hypogammaglobulinemia, and response to CsA are all nonspecific, and because T-LGL immunophenotyping, TCR clonality testing, and STAT3/STAT5B testing were not performed in this case, T-LGLL cannot currently be definitively diagnosed. Nevertheless, the possibility of an abnormal T-LGL-like CD8^+^ T-cell clone should still be considered, as it may also explain the persistence of agranulocytosis after surgery.

### 3.3 Clinical significance of surgery for thymoma-associated hematologic disorders

After thymoma resection in this case, the absolute reticulocyte count increased from 5.0×10^9^/L to 213.0×10^9^/L without further red blood cell transfusions, and the Hb level also increased gradually. More importantly, this trend emerged before the patient began regular CsA therapy, suggesting that thymoma resection may have been an important factor in the improvement of PRCA. Previous studies likewise support a therapeutic role for surgery in some patients with thymoma-associated PRCA (1–4, 19).

The hematologic benefits of surgery are not limited to PRCA. A previous report described a patient with thymoma accompanied by neutropenia and thrombocytopenia whose granulocyte and platelet counts recovered within several days after thymectomy (20). Hematologic remission has also been achieved in patients with thymoma-associated autoimmune hemolytic anemia after surgery combined with glucocorticoid therapy (21). From an immunologic perspective, thymoma resection can remove the abnormal thymic microenvironment, reduce the ongoing generation and peripheral export of autoreactive T cells, and potentially improve Treg-related immune tolerance, thereby alleviating the persistent attack on bone marrow hematopoietic cells (5, 12–14). Therefore, in patients with thymoma-associated hematologic paraneoplastic syndromes whose tumors are technically resectable and whose perioperative risk is acceptable, surgery provides value not only for local tumor control but also potentially for promoting hematopoietic recovery and should be regarded as an important component of comprehensive treatment.

Repeat surgery may also be valuable for recurrent thymoma. Such recurrences are often confined to the local or pleural region and tend to progress relatively slowly, making repeat resection feasible. A retrospective analysis of a Japanese nationwide database identified 420 postoperative recurrences among 2,835 patients with thymic epithelial tumors who initially underwent surgery. Of these, 162 patients underwent repeat surgery, with 5- and 10-year post-recurrence survival rates of 82.7% and 68.2%, respectively; among 243 patients who received nonsurgical treatment, the corresponding survival rates were 43.5% and 25.4% (22). The benefit of repeat surgery was more pronounced in patients with a longer interval to recurrence, localized lesions, and a reasonable prospect of complete or near-complete resection (22). Therefore, repeat surgery should be considered after careful assessment of lesion resectability and surgical risk.

It may be further hypothesized that, for both primary and recurrent thymoma, early resection could terminate the ongoing generation and peripheral export of autoreactive T cells at an earlier stage, reduce immune attack on the bone marrow, and decrease the likelihood of continued expansion of abnormal T-cell clones with specific TCR rearrangements and T-LGL-like CD8^+^ T cells (5, 7–9, 13, 14). This might reduce the risks of postoperative agranulocytosis, T-LGLL, and other hematologic disorders, although this immunologic benefit requires verification in further studies. It should be noted that surgery mainly removes the source of ongoing abnormal T-cell generation and may not immediately eliminate T cells that have already entered the peripheral blood or bone marrow and acquired long-term survival or undergone clonal expansion (7–9, 17, 23). Therefore, blood counts and relevant immunologic parameters should continue to be monitored serially after surgery, and adjunctive immunosuppressive therapy should be considered when necessary.

### 3.4 Immunosuppressive therapy after thymoma resection

In the present case, after regular oral CsA treatment at 200 mg/day postoperatively, the WBC and ANC still showed no clear improvement, and intermittent G-CSF remained necessary. After the dose was increased to 300 mg/day and drug exposure rose, the WBC and ANC increased overall without further G-CSF administration, suggesting that sustained immunosuppressive therapy achieving an appropriate level of drug exposure may facilitate granulopoietic recovery. CsA acts mainly by inhibiting activated T cells but does not selectively target CD8^+^ T cells. After binding to cyclophilin, CsA inhibits calcineurin, blocks the dephosphorylation and nuclear translocation of nuclear factor of activated T cells (NFAT), and downregulates transcription of cytokines such as interleukin-2 (IL-2), thereby suppressing TCR-mediated T-cell activation, proliferation, and effector function (24). Given the potential pathogenic role of abnormally activated CD8^+^ T cells, CsA may alleviate immune-mediated injury to granulocytic progenitors by suppressing their persistent activation and clonal expansion (6, 16, 17).

Previous case reports likewise support the therapeutic role of CsA. Kobayashi et al. reported a 63-year-old man with thymoma and pure white cell aplasia. Granulocytic cells reappeared in the bone marrow on day 13 after CsA initiation, peripheral neutrophils began to increase on day 18, and thymectomy was performed 3 months later; long-term remission was maintained even after CsA discontinuation (17). Fukushima et al. reported a 33-year-old man who developed PRCA after treatment for pleural recurrence of thymoma. Tacrolimus was ineffective, but approximately 2 weeks after switching to CsA 250 mg/day, the reticulocyte count began to increase and the anemia subsequently improved. The CsA trough concentration was maintained at 100–150 ng/mL, with no obvious treatment-related adverse events (25). Together with the present case, these reports suggest that when immune-mediated bone marrow injury persists after thymoma resection or other antitumor treatment, CsA may be an important therapeutic option for promoting hematopoietic recovery (2–4, 17, 25).

The present case also highlights the importance of therapeutic drug monitoring for assessing CsA exposure and guiding dose adjustment. At an oral CsA dose of 200 mg/day, the whole-blood concentration was only 62.23 ng/mL, and neutropenia showed no clear improvement. After the dose was increased to 300 mg/day, the concentration rose to 173.39 ng/mL, followed by an overall increase in the WBC and ANC. Previous PRCA studies have generally maintained steady-state whole-blood CsA trough concentrations at 150–250 ng/mL, and the prescribing information also lists ≥100 ng/mL as a reference value for effective exposure (26–28). However, direct studies of the concentration-response relationship in thymoma-associated agranulocytosis are lacking, and 100 ng/mL therefore cannot be regarded as a definitive efficacy threshold.

In addition to CsA, other immunosuppressive agents may be considered as alternatives in selected situations. Glucocorticoids can suppress T-cell activation and inflammatory cytokine production, but infection and metabolic risks must be comprehensively assessed. Because the present patient had previously developed glucocorticoid-associated hyperglycemia, their use would require particular caution. Sirolimus can inhibit the mTOR pathway; given the possible activation of the PI3K-AKT-mTOR/T-bet pathway in bone marrow CD8^+^ T cells from patients with PRCA, there is a mechanistic rationale for its use in patients who respond poorly to or cannot tolerate CsA (16, 29). If evaluation of large granular lymphocyte morphology, flow-cytometric immunophenotyping, TCR gene rearrangement, and STAT3/STAT5B testing suggests a persistent T-LGL-like clone or features consistent with T-LGLL, methotrexate or cyclophosphamide may be considered after hematology consultation (30). If multilineage cytopenia or aplastic anemia-like changes develop, intensified immunosuppressive regimens such as antithymocyte globulin combined with CsA may also be considered on the basis of experience in related conditions (31). These options are based mainly on relevant immune mechanisms and treatment experience in similar diseases, and their efficacy in thymoma-associated agranulocytosis remains insufficiently validated. Treatment should therefore be individualized according to bone marrow findings, T-cell immunophenotype and clonality, infection status, and organ function.

In patients with thymoma and hematologic disorders, early and consistent immunosuppression may be considered when infection is controlled, hepatic and renal function permit, and treatment is tolerated, with dose adjustment guided by drug levels and adverse events. Early in this case, poor adherence limited the actual CsA dose to 75 mg/day, and neither anemia nor neutropenia improved. Even after surgery, the WBC and ANC increased only after regular treatment and higher drug exposure. Thus, ensuring adherence and promptly achieving appropriate exposure may help control persistent immune-mediated granulopoietic injury and shorten agranulocytosis and G-CSF dependence. However, this potential benefit of earlier treatment is a retrospective inference from this single case and requires validation.

### 3.5 Limitations

This study has several limitations. First, this is a single-case observation with a relatively limited follow-up period; therefore, the long-term stability of hematopoietic recovery and the long-term hematologic outcomes require further observation. Second, detailed T-cell immunophenotyping, T-cell receptor clonality analysis, and STAT3/STAT5B testing were not performed. Although previous studies and the clinical course of the present case support the possible involvement of aberrant cytotoxic T cells in immune-mediated hematopoietic injury, direct evidence confirming their specific role is lacking. In addition, the coexistence of MG suggests a broader background of thymoma-associated systemic autoimmunity, further complicating attribution of the underlying immune mechanism. Third, although erythroid recovery showed a close temporal association with thymoma resection and granulopoietic improvement with increased CsA exposure, and previous studies also support their potential therapeutic roles, the causal contribution of each intervention to hematopoietic recovery cannot be fully established on the basis of a single case. Therefore, this case provides clinically valuable insights into the potential immune mechanisms and therapeutic strategies, which warrant further validation in larger cohorts with longer follow-up and more comprehensive immunologic evaluation.

## 4 Conclusion

When thymoma is accompanied by hematologic abnormalities, immune-mediated injury to bone marrow hematopoiesis should be considered. In the present case, erythropoiesis gradually recovered after resection of the recurrent lesion, whereas persistent agranulocytosis improved only after CsA was administered regularly and drug exposure was increased, suggesting that treatment should address both the “source” and the “persistence” of the abnormal immune response. For patients with localized lesions that are amenable to complete resection and for whom perioperative risk is acceptable, surgery can control the tumor and may also promote hematopoietic recovery by reducing the continued generation and export of abnormal T cells; however, it is unlikely to immediately eliminate abnormal T cells that have already entered the peripheral blood or bone marrow and continue to survive, and persistent hematologic abnormalities may therefore remain after surgery. A multidisciplinary management approach is therefore warranted, with serial monitoring of peripheral blood counts and repeat bone marrow examination when necessary. For persistent or recurrent immune-mediated hematopoietic injury after surgery, the immunophenotype and clonality of cytotoxic T cells may be evaluated; when infection is controlled and organ function permits, immunosuppressive therapy such as CsA should be initiated early and administered regularly, with the regimen adjusted according to therapeutic drug concentrations and hepatic and renal function, to achieve more stable hematopoietic recovery and improve treatment safety.

## Data Availability

The original contributions presented in the study are included in the article/**Supplementary Material**. Further inquiries can be directed to the corresponding author.
