# Supplementary material for: Recurrent thymoma-associated PRCA with persistent agranulocytosis: a case report and therapeutic considerations
Source: Front Oncol. 2026 Sep 28;16:1930291. doi: 10.3389/fonc.2026.1930291 (PMC13655922; doi:10.3389/fonc.2026.1930291)
Supplement: Supplementary file 1 [file DataSheet1.pdf]

Supplementary Figure S1.

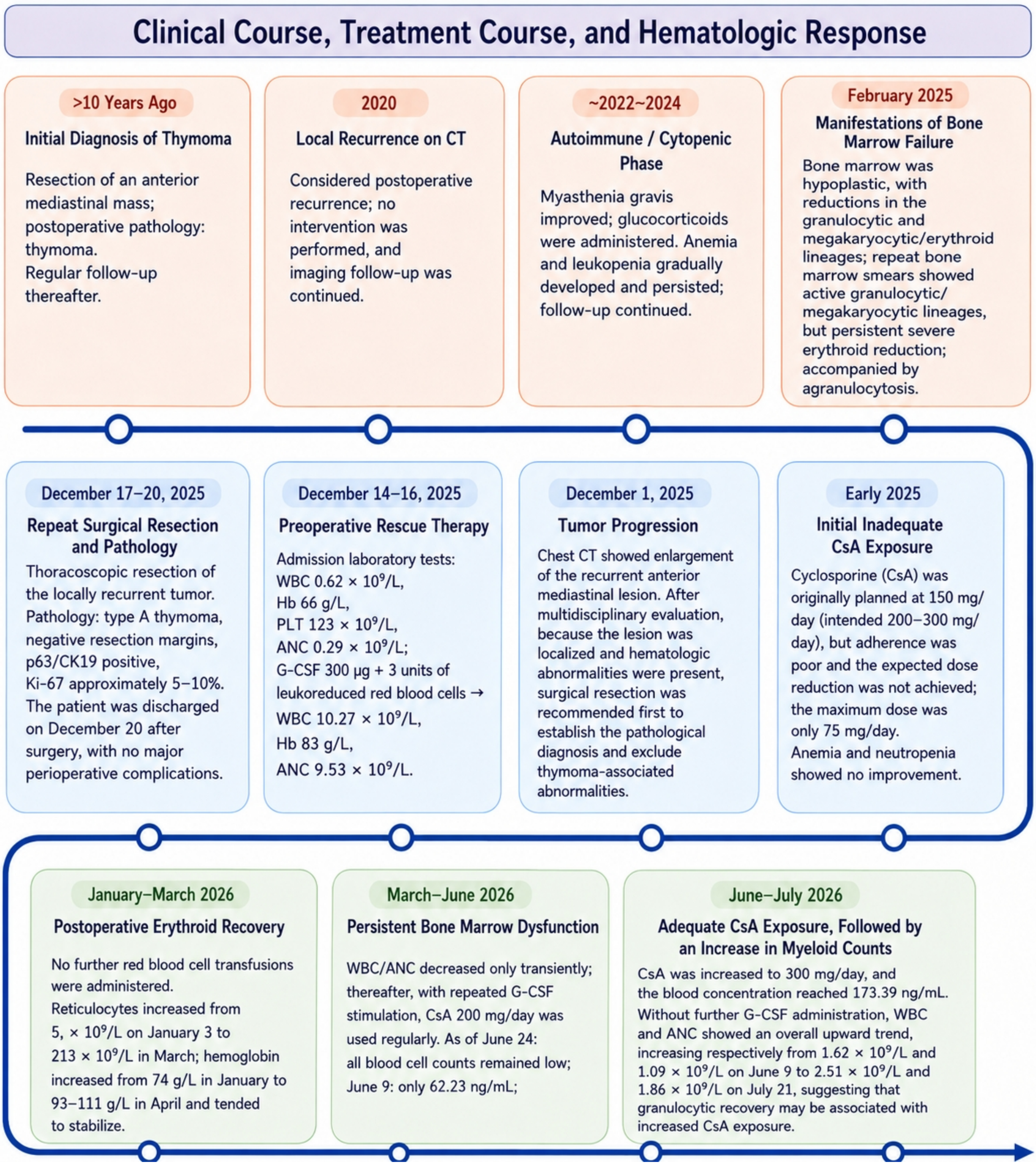

Supplementary Table S1.

Time point	WBC ($\times 10^9$/L)	Hb (g/L)	PLT ($\times 10^9$/L)	ANC ($\times 10^9$/L)	ALC ($\times 10^9$/L)	RET% / RET#	Globulin (g/L)	Treatment / clinical interpretation
2025-12-15 (Admission; before treatment)	0.62	66	123	0.29	0.20	NR	20.4	Severe agranulocytosis and anemia at admission, before preoperative G-CSF and red blood cell transfusion.
2025-12-16 (After preoperative support)	10.27	83	151	9.53	0.41	NR	NR	After preoperative G-CSF 300 μg/day and 2 U of leukoreduced packed red blood cells; transient hematologic correction before surgery.
2025-12-18 (Postoperative day 1)	7.44	96	142	7.00	0.23	NR	19.5	One day after thoracoscopic resection on 17 December; the granulocyte increase remained confounded by recent G-CSF.
2026-01-03 (Postoperative follow-up)	2.15	74	180	1.67	0.31	0.20% / 5.00$\times 10^9$/L	22.1	Early postoperative decline in WBC and ANC, with persistent reticulocytopenia and anemia.
2026-03-02 (Follow-up)	1.31	75	140	0.88	0.29	2.60% / 64.00$\times 10^9$/L	17.9	Reticulocyte recovery became evident, whereas neutropenia persisted.
2026-03-13 (Follow-up)	1.78	82	145	1.16	0.44	8.48% / 213.00$\times 10^9$/L	18.1	Marked reticulocyte response and rising hemoglobin without further red blood cell transfusion.
2026-03-24 (Regular CsA initiated)	0.98	88	137	0.55	0.30	7.68% / 197.00$\times 10^9$/L	18.9	Recurrent agranulocytosis; G-CSF was administered, and regular CsA 200 mg/day in two divided doses was initiated.
2026-04-07 (Follow-up)	1.00	101	166	0.43	0.39	1.71% / 48.90$\times 10^9$/L	NR	Persistent agranulocytosis despite CsA 200 mg/day; G-CSF was administered.
2026-04-09 (Two days after G-CSF)	4.16	94	146	3.46	0.39	NR	NR	Transient WBC and ANC rebound after the G-CSF administration on 7 April.
2026-04-21 (Follow-up)	1.72	101	159	1.17	0.40	3.64% / 103.00$\times 10^9$/L	20.5	Myeloid counts declined again after the transient G-CSF response; erythroid recovery persisted.
2026-05-12 (Follow-up)	0.76	103	128	0.30	0.33	1.81% / 52.00$\times 10^9$/L	21.0	Recurrent severe agranulocytosis; G-CSF was administered while CsA 200 mg/day was continued.
2026-05-28 (Follow-up)	1.63	93	133	1.24	0.28	2.27% / 60.00$\times 10^9$/L	19.5	Counts rose from the 12 May nadir but remained unstable; transfusion independence was maintained.

Time point	WBC (×10⁹/L)	Hb (g/L)	PLT (×10⁹/L)	ANC (×10⁹/L)	ALC (×10⁹/L)	RET% / RET#	Globulin (g/L)	Treatment / clinical interpretation
2026-06-09 (CsA monitoring and dose escalation)	1.62	111	165	1.09	0.36	3.42% / 104.00×10⁹/L	24.1	Whole-blood CsA concentration was 62.23 ng/mL on 200 mg/day; the dose was increased to 300 mg/day in two divided doses.
2026-06-23 (Follow-up after dose escalation)	1.90	102	141	1.25	0.48	2.17% / 62.00×10⁹/L	22.3	Early follow-up on CsA 300 mg/day; no further G-CSF was documented after 12 May.
2026-07-07 (CsA concentration reassessment)	1.71	101	131	1.20	0.37	3.42% / 99.00×10⁹/L	24.1	Whole-blood CsA concentration increased to 173.39 ng/mL on 300 mg/day.
2026-07-14 (Follow-up)	2.31	96	127	1.63	0.43	NR	NR	WBC and ANC continued to rise without further documented G-CSF.
2026-07-21 (Latest available follow-up)	2.51	102	147	1.86	0.46	NR	22.7	Continued myeloid improvement on CsA 300 mg/day; transfusion independence was maintained, and no thymoma recurrence was reported.

Abbreviations: ALC, absolute lymphocyte count; ANC, absolute neutrophil count; CsA, cyclosporine A; G-CSF, granulocyte colony-stimulating factor; Hb, hemoglobin; NR, not reported in the supplied source materials; PLT, platelet count; RET#, absolute reticulocyte count; RET%, reticulocyte percentage; WBC, white blood cell count.

Outpatient G-CSF was explicitly documented on 24 March, 7 April, and 12 May 2026, but the doses were not stated in the supplied records. Preoperative G-CSF was documented as 300 µg/day.
